# Diagnostic Accuracy of Natriuretic Peptides for Heart Failure in Patients with Pleural Effusion: A Systematic Review and Updated Meta-Analysis

**DOI:** 10.1371/journal.pone.0134376

**Published:** 2015-08-05

**Authors:** Zhi-Jun Han, Xiao-Dan Wu, Juan-Juan Cheng, Shi-Di Zhao, Ming-Zhu Gao, Hong-Yu Huang, Bing Gu, Ping Ma, Yan Chen, Jun-Hong Wang, Cheng-Jian Yang, Zi-He Yan

**Affiliations:** 1 Department of Laboratory Medicine, Wuxi Second People’s Hospital of Nanjing Medical University, Wuxi, Jiangsu, China; 2 Department of Cardiology, Wuxi Second People’s Hospital of Nanjing Medical University, Wuxi, Jiangsu, China; 3 Department of Laboratory Medicine, The Affiliated Hospital of Xuzhou Medical College, Xuzhou, Jiangsu, China; 4 Emergency Center, The First Affiliated Hospital of Nanjing Medical University, Nanjing, Jiangsu, China; 5 Department of Gerontology, The First Affiliated Hospital of Nanjing Medical University, Nanjing, Jiangsu, China; Scuola Superiore Sant'Anna, ITALY

## Abstract

**Background:**

Previous studies have reported that natriuretic peptides in the blood and pleural fluid (PF) are effective diagnostic markers for heart failure (HF). These natriuretic peptides include N-terminal pro-brain natriuretic peptide (NT-proBNP), brain natriuretic peptide (BNP), and midregion pro-atrial natriuretic peptide (MR-proANP). This systematic review and meta-analysis evaluates the diagnostic accuracy of blood and PF natriuretic peptides for HF in patients with pleural effusion.

**Methods:**

PubMed and EMBASE databases were searched to identify articles published in English that investigated the diagnostic accuracy of BNP, NT-proBNP, and MR-proANP for HF. The last search was performed on 9 October 2014. The quality of the eligible studies was assessed using the revised Quality Assessment of Diagnostic Accuracy Studies tool. The diagnostic performance characteristics (sensitivity, specificity, and other measures of accuracy) were pooled and examined using a bivariate model.

**Results:**

In total, 14 studies were included in the meta-analysis, including 12 studies reporting the diagnostic accuracy of PF NT-proBNP and 4 studies evaluating blood NT-proBNP. The summary estimates of PF NT-proBNP for HF had a diagnostic sensitivity of 0.94 (95% confidence interval [CI]: 0.90–0.96), specificity of 0.91 (95% CI: 0.86–0.95), positive likelihood ratio of 10.9 (95% CI: 6.4–18.6), negative likelihood ratio of 0.07 (95% CI: 0.04–0.12), and diagnostic odds ratio of 157 (95% CI: 57–430). The overall sensitivity of blood NT-proBNP for diagnosis of HF was 0.92 (95% CI: 0.86–0.95), with a specificity of 0.88 (95% CI: 0.77–0.94), positive likelihood ratio of 7.8 (95% CI: 3.7–16.3), negative likelihood ratio of 0.10 (95% CI: 0.06–0.16), and diagnostic odds ratio of 81 (95% CI: 27–241). The diagnostic accuracy of PF MR-proANP and blood and PF BNP was not analyzed due to the small number of related studies.

**Conclusions:**

BNP, NT-proBNP, and MR-proANP, either in blood or PF, are effective tools for diagnosis of HF. Additional studies are needed to rigorously evaluate the diagnostic accuracy of PF and blood MR-proANP and BNP for the diagnosis of HF.

## Introduction

The presence of pleural fluid (PF) is commonly encountered in clinical practice. The development of PF is associated with a broad range of etiologies, such as heart failure (HF), tuberculosis, malignancy, and pulmonary embolism [1]. PF is generally categorized as either transudate, which is usually caused by HF (80% of cases) or cirrhosis (20% of cases), or exudate, which is generally caused by localized inflammatory disease such as infection or malignancy. During the past decades, the criteria established by Light et al. [2] have been widely used to differentiate exudates from transudates. Although these criteria have high sensitivity in the identification exudates, they have low specificity [3], and approximately 25% of transudates are erroneously identified as exudates according to the Light criteria [4].

Among patients with pleural effusion, the diagnosis of HF typically depends on the medical history, signs, and symptoms, as well as the presence of specific biomarkers. As some of the symptoms and signs (e.g., dyspnea and fatigue) can also be observed in non-HF patients, biomarkers can be more valuable for the differentiation of pleural effusions resulting from HF from those of other causes.

Natriuretic peptides constitute a family of hormones that are produced and secreted by the heart muscle in response to increased tension or stretching and other stimulatory factors, such as other peptides and hormones (e.g., glucocorticoids and thyroid hormones), biologic substances (e.g., nitric oxide), and cytokines (e.g., interleukin-1 and interleukin-6) [5, 6]. Accumulated evidence shows that serum or plasma levels of three natriuretic peptides, namely brain natriuretic peptide (BNP), amino-terminal proBNP (NT-proBNP), and midregion pro-atrial natriuretic peptide (MR-proANP), are powerful diagnostic tools for HF [7–13]. Three meta-analyses performed in 2010 and 2011 found that an increased level of NT-proBNP in PF has a high diagnostic accuracy for HF [14–16]. However, additional studies have since been published on this topic, and an updated meta-analysis is necessary to estimate the diagnostic accuracy of PF NT-proBNP for HF. Furthermore, two other natriuretic peptides in addition to PF NT-proBNP, namely blood and PF BNP and MR-proANP, have also been described as effective diagnostic markers for HF. Therefore, we performed the present systematic review and updated meta-analysis to establish the overall diagnostic accuracy of these natriuretic peptides for HF in patients with PF.

## Materials and Methods

### Search strategy and study selection

This meta-analysis was performed and reported in accordance with the Preferred Reporting Items for Systematic Reviews and Meta-Analyses (PRISMA) guidelines (S1 Checklist. PRISMA checklist) [17]. The EMBASE and PubMed databases were searched to identify potentially eligible studies published in English through 9 October 2014. The keywords and algorithm for the literature search for PubMed were as follows: (“natriuretic peptide” OR “natriuretic peptides” OR “BNP” OR “nt-pro-bnp” OR “ANP” OR “mr-pro-anp”) AND (“heart failure” OR “cardiac failure” OR “cardiac”) AND (“pleural fluid” OR “pleural fluids” OR “pleural effusion” OR “pleural effusions”). A similar strategy was used when searching the EMBASE database. A manual search was also performed using the references listed in the retrieved articles.

The inclusion criteria for the present study were: 1) evaluation of the diagnostic accuracy of blood or PF natriuretic peptides (BNP, NT-proBNP, and/or MR-proANP) for the diagnosis of HF; 2) a sample of > 10 patients with or without HF because small samples can introduce marked bias in estimations of sensitivity and specificity [18]; 3) reported sensitivity and specificity of natriuretic peptides or the ability to obtain these parameters from the receiver operating characteristic (ROC) curve; and 4) reported sample sizes of the patients with and without HF. Studies not published in English, conference abstracts, letters to the editor, and animal studies were excluded from the analysis. When duplicate reports were encountered, only the study with more detailed information was included.

### Data extraction and quality assessment

Two investigators independently retrieved the scientific literature. The titles and abstracts of all potentially relevant studies were reviewed to identify any eligible studies. A full-text review was performed when necessary. Any disagreements regarding study eligibility were resolved by discussion and consensus.

The two investigators independently performed the data extraction and quality assessment. The following data were extracted from each eligible study: name of the first author, publication year, source of participants, sample size, control component, reference used for HF diagnosis, type of data collection (prospective or retrospective), assay used for natriuretic peptides detection, and area under the ROC curve (AUC). A 2 × 2 table was constructed, and the true-positive, false-positive, false-negative, and true-negative rates were calculated.

Two investigators independently assessed the quality of all included studies using the revised Quality Assessment of Diagnostic Accuracy Studies (QUADAS-2) tool [19]. The corresponding authors of the eligible studies were not contacted to obtain detailed design information if the necessary data were not reported in the full text; in such cases, the corresponding items or domain in the QUADAS-2 were labeled “unknown.” Any disagreement regarding the quality assessment was resolved by consensus.

### Statistical analysis

The overall diagnostic sensitivity and specificity of each natriuretic peptide for HF were pooled using a bivariate model [20]. Forest plots were used to graphically depict the heterogeneity across all eligible studies as well as the overall diagnostic sensitivity and specificity. Cochrane’s *Q* and the inconsistency index (*I* ^*2*^) were calculated to determine the degree and significance of heterogeneity across all eligible studies [21]. We also explored the degree to which heterogeneity could be explained by the threshold effect. The Fagan method [22] was used to calculate the post-test probability, and the prevalence of HF among all patients was assumed to be the pretest probability of HF. Funnel plots and Deeks’ test was performed to determine the presence of publication bias [23]. All analyses were performed using STATA 12.0 (Stata Corp LP, College Station, TX, USA), and the midas command was used for all statistical analyses [24].

## Results

### Summary of eligible studies

**Fig 1:**
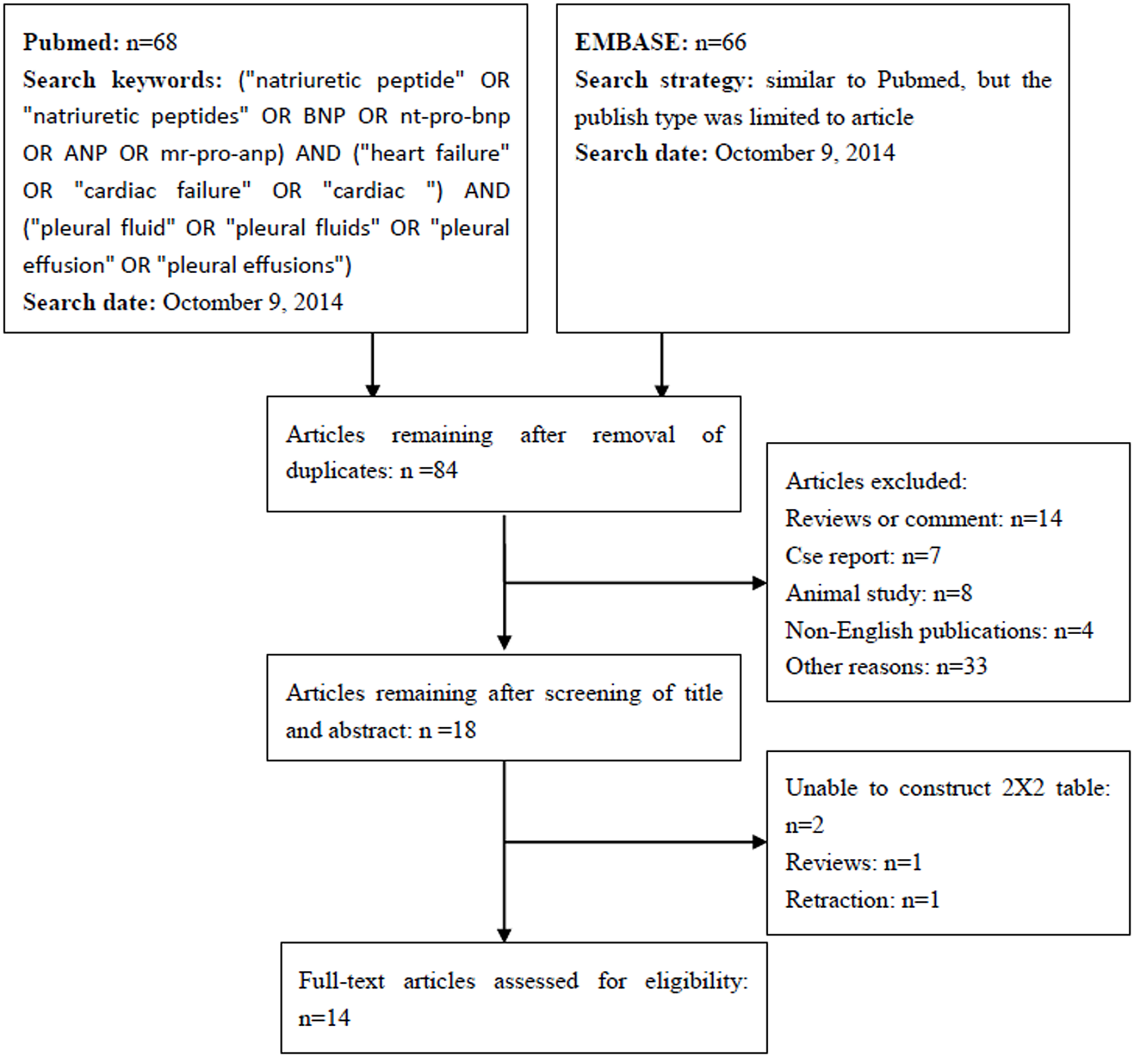
Flow chart illustrating the systematic literature search and study selection process.

In total, 14 studies involving 599 patients with HF and 1055 patients without HF were included in the present work [25–38]. A flow chart depicting the study selection process is shown in Fig 1. A summary of the most pertinent details of the eligible studies is shown in Table 1. The sample size of these 14 studies ranged from 28 to 398. Half of the studies were prospective [25, 27, 32, 34–36, 38], five were retrospective [26, 28–31], and the remaining two did not report their design characteristics [33, 37]. The control groups generally comprised patients with malignancy, tuberculosis, parapneumonic effusion, pulmonary embolism, hepatic hydrothorax, postcardiac injury syndrome, collagen disease, and other conditions.

**Table 1:** Summary of eligible studies.

First author	Year	Country	No.	HF/non-HF	Characteristics of controls	Reference	Data collection
Cincin [25]	2013	Turkey	66	21/45	M, PP, empyema, TB, renal failure	Clinical	Prospective
Porcel [26]	2013	Spain	185	95/90	M, TB, PP, HH, PE, pericardial disease	Framingham score	Retrospective
Valdes [27]	2011	Spain	398	94/304	LC, NS, M, PP, TB, miscellaneous	Clinical	Prospective
Marinho [28]	2011	Brazil	77	34/43	M, TB, HH	Clinical	Unknown
Long [29]	2010	USA	80	20/60	PCABG, pneumonia, M	Clinical	Retrospective
Porcel [30]	2009	Spain	181	90/91	HH, M, PP, TB, PE, hemothorax, Dressler syndrome, drug-induced	Framingham score	Retrospective
Bayram [31]	2009	Turkey	133	51/82	LC, NS, TB, PE, M, PP	Clinical	Prospective
Han [32]	2008	Korea	240	82/158	LC, TB, PP, M	Clinical	Prospective
Liao [33]	2008	China	40	10/30	PCABG, PE, M	Clinical	Unknown
Porcel [34]	2007	Spain	93	53/40	M, LC, TB, PP, NS, PE, PCIS, hypoalbuminemia, hemothorax, paradoxical response to antituberculous therapy, atelectasis, postabdominal surgical procedure, uremia	Clinical	Prospective
Kolditz [35]	2006	Germany	93	25/68	M, PP, empyema, acute pleuritis, PCIS, after local surgery, CD, TB, chylothorax	Clinical	Prospective
Gegenhuber [36]	2005	Austria	57	31/26	M, infective diseases, LC, TB, renal failure	Clinical	Prospective
Porcel [37]	2004	Spain	117	44/57	M, TB, PP, HH, PE	Clinical	Unknown
Tomcsanyi [38]	2004	Hungary	28	14/14	M, TB, collagen disease, PCIS	Framingham score	Prospective

M: malignancy, TB: tuberculosis, PP: parapneumonic effusion, PE: pulmonary embolism, LC: liver cirrhosis, HF: heart failure, HH: hepatic hydrothorax, NS: nephrotic syndrome, PCABG: postcoronary artery bypass grafting, PCIS: postcardiac injury syndrome, CD: collagen disease

### Quality assessment of eligible studies

Table 2 presents the design quality of all eligible studies as assessed by the QUADAS-2. In general, the patient selection, index test, and reference standard domains for application concerns were labeled “low-risk” because the enrolled patients, index test used for evaluation, and reference standard used were well matched to the review question in all studies. In three studies, the risk of bias associated with patient selection was determined to be high because the patients were not consecutively or randomly enrolled [26, 29, 38]. One study [37] was labeled “unknown” because the author did not report the method of patient recruitment. The index test domain of nine studies was labeled “high” because, although the sample sizes were small, the threshold was not prespecified [25, 29, 31, 33, 35–38]. This domain in one study was labeled “unknown” because the author did not report whether the index test results were interpreted without knowledge of the reference standard results [32, 34]. The reference standard domain of two studies was labeled “unknown” because the authors did not report whether the clinicians were blinded to the index test results when they established the diagnosis [29, 38]. The flow and timing domain for all studies except two [28, 34] was labeled “unknown” because the author did not report whether all enrolled patients received the same reference standard; therefore, neither partial nor differential verification bias could be completely excluded.

**Table 2:** Quality assessment of eligible studies by QUADAS-2.

Study	Risk of bias				Applicability concerns		
	Patient selection	Index test	Reference standard	Flow and timing	Patient selection	Index test	Reference standard
Cincin [25]	Low	High	Low	Unknown	Low	Low	Low
Porcel [26]	High	Low	Low	Unknown	Low	Low	Low
Valdes [27]	Low	Low	Low	Unknown	Low	Low	Low
Marinho [28]	Low	Low	Low	Low	Low	Low	Low
Long [29]	High	High	Unknown	Unknown	Low	Low	Low
Porcel [30]	Low	Low	Low	Unknown	Low	Low	Low
Bayram [31]	Low	High	Low	Unknown	Low	Low	Low
Han [32]	Low	Unknown	Low	Unknown	Low	Low	Low
Liao [33]	Low	High	Low	Unknown	Low	Low	Low
Porcel [34]	Low	High	Low	Low	Low	Low	Low
Kolditz [35]	Low	High	Low	Unknown	Low	Low	Low
Gegenhuber [36]	Low	High	Low	Unknown	Low	Low	Low
Porcel [37]	Unknown	High	Low	Unknown	Low	Low	Low
Tomcsanyi [38]	High	High	Unknown	Unknown	Low	Low	Low

### Diagnostic accuracy of natriuretic peptides for HF in patients with PF

As shown in Table 3, 13 studies evaluated the diagnostic accuracy of PF NT-proBNP for HF [25–27, 29–38], three studies investigated PF BNP [28–30], and one study investigated PF MR-proANP [26]. Two studies investigated the diagnostic accuracy of blood BNP [28, 36], and four studies evaluated blood NT-proBNP [27, 31, 34, 35].

**Table 3:** Diagnostic characteristics reported by eligible studies.

Biomarkers	Study	Assay	AUC	Threshold (ng/L)	TP	FP	FN	TN	Sensitivity (95% CI)	Specificity (95% CI)
**PF NT-proBNP**										
	Cincin [25]	Roche	0.93 (0.86–1.00)	2300	20	6	1	39	0.958 (–)	0.857 (–)
	Porcel [26]	Cobas	0.94 (0.89–0.97)	1700	87	16	8	74	0.92 (0.84–0.96)	0.82 (0.73–0.89)
	Valdes [27]	Roche	0.89 (0.86–0.92)	1409	80	62	14	242	0.85 (0.76–0.92)	0.80 (0.75–0.84)
	Long [29]	Biomedica	0.84 (0.72–0.95)	2000	16	16	4	44	0.80 (0.58–0.92)	0.73 (0.61–0.83)
	Porcel [30]	Roche	0.96 (0.94–0.99)	1500	84	10	6	81	0.93 (0.86–0.98)	0.89 (0.81–0.95)
	Bayram [31]	Roche	0.97 (–)	925	48	4	3	78	0.94 (0.87–0.97)	0.95 (0.88–0.99)
	Han [32]	Roche	1.00 (0.99–1.00)	1714	81	1	1	157	0.99 (0.93–1.00)	0.99 (0.96–1.00)
	Liao [33]	ALPCO	0.99 (0.97–1.00)	2220	10	1	0	29	1.00 (–)	0.97 (–)
	Porcel [34]	Roche	0.93 (0.87–0.99)	1500	49	5	4	35	0.92 (0.84–1.00)	0.87 (0.76–0.99)
	Kolditz [35]	Roche	0.98 (0.96–1.00)	4000	23	5	2	63	0.92 (0.74–0.99)	0.93 (0.84–0.98)
	Porcel [37]	Roche	0.97 (0.94–1.00)	1500	40	5	4	68	0.91 (0.81–1.00)	0.93 (0.87–1.00)
	Tomcsanyi [38]	Roche	NR	599	14	0	0	14	1.00 (–)	1.00 (–)
**PF BNP**										
	Marinho [28]	Advia	0.95 (0.87–0.99)	127	33	5	1	38	0.97 (0.85–1.00)	0.88 (0.74–0.96)
	Long [29]	Peninsula Laboratories	0.70 (0.57–0.83)	NR	14	25	6	35	0.70 (–)	0.58 (–)
	Porcel [30]	Advia	0.90 (0.86–0.95)	115	67	7	23	84	0.74 (0.64–0.83)	0.92 (0.85–0.97)
**Blood NT-proBNP**										
	Valdes [27]	Roche	0.89 (0.84–0.92)	748	84	82	10	222	0.90 (0.80–0.96)	0.73 (0.66–0.78)
	Porcel [34]	Roche	0.92 (0.85–0.98)	1500	49	6	4	34	0.92 (0.84–1.00)	0.85 (0.73–0.97)
	Kolditz [35]	Roche	0.98 (0.96–1.00)	4000	22	5	3	63	0.88 (0.69–0.97)	0.93 (0.84–0.98)
	Bayram [31]	Roche	0.97 (–)	1040	48	4	3	78	0.94 (0.88–0.96)	0.95 (0.88–0.99)
**Blood BNP**										
	Marinho [28]	Advia	0.99 (0.93–1.00)	132	33	1	1	42	0.97 (0.85–1.00)	0.97 (0.87–1.00)
	Gegenhuber [36]	Abbott	0.98 (0.89–1.00)	520	30	3	1	23	0.97 (0.83–1.00)	0.89 (0.70–0.97)
**PF MR-proANP**										
	Porcel [26]	BRAHMS	0.92 (0.87–0.95)	260 pmol/L	80	15	15	75	0.84 (0.75–0.91)	0.83 (0.74–0.90)

AUC: area under the receiver operating characteristic curve, TP: true-positive rate, FP: false-positive rate, FN: false-negative rate, TN: true-negative rate, 95%CI: 95% confidence interval, PF: pleural fluid, NR: not reported, NT-proBNP: N-terminal pro-brain natriuretic peptide, BNP: brain natriuretic peptide, MR-proANP: midregion pro-atrial natriuretic peptide

**Fig 2:**
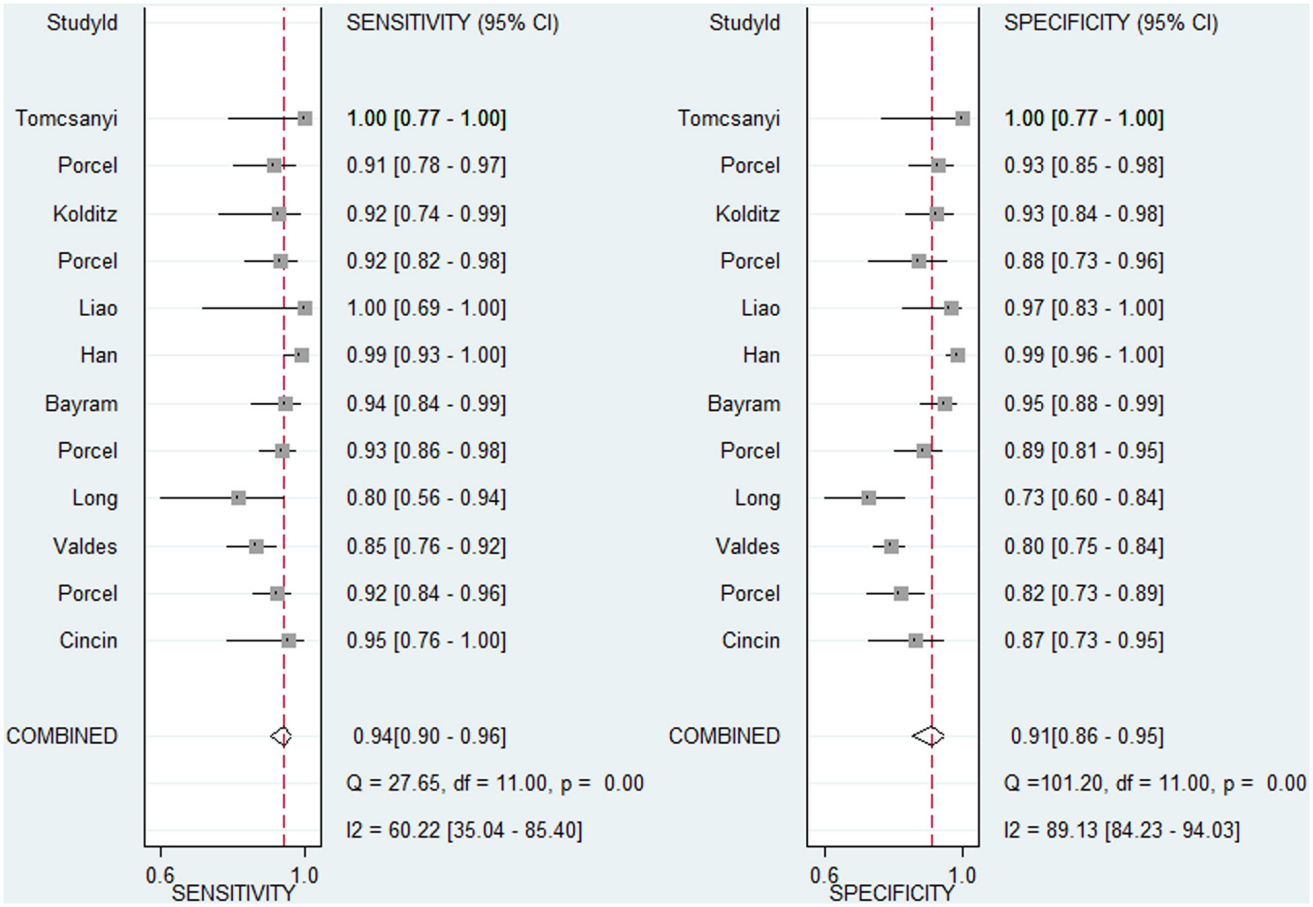
Forest plot of the estimates of sensitivity and specificity for pleural fluid N-terminal pro-brain natriuretic peptide in the diagnosis of heart failure.

Because the diagnostic accuracy of blood BNP, PF BNP, and PF MR-proANP for HF was investigated in only two, three, and one study, respectively, we only evaluated the diagnostic accuracy of PF and blood NT-proBNP for HF in the present meta-analysis. These results are presented in Table 4. The overall sensitivity and specificity of PF NT-proBNP for diagnosis of HF were 0.94 and 0.91, respectively. The *I* ^*2*^ for sensitivity and specificity were 60.22 (95% confidence interval [CI]: 35.04–85.40) and 89.13 (95% CI: 84.23–94.03), respectively. Bivariate model analysis showed that this heterogeneity was completely (100%) explained by the threshold effect. Fig 2 graphically depicts the overall diagnostic sensitivity and specificity as well as the heterogeneity across all 12 studies that investigated the diagnostic accuracy of PF NT-proBNP for HF. The overall sensitivity and specificity of blood NT-proBNP for diagnosis of HF were 0.92 and 0.88, respectively. The *I* ^*2*^ for sensitivity and specificity were 17.48 (95% CI: 0.00–100.00) and 94.58 (95%CI: 90.85–98.32), respectively. Bivariate model analysis also showed that this heterogeneity was completely (100%) explained by the threshold effect. Considering that the heterogeneity across the studies could be completely explained by the threshold effect, neither subgroup analysis nor meta-regression was performed.

**Table 4:** Overall diagnostic accuracy of blood and pleural fluid NT-proBNP for heart failure.

NT-proBNP	No. of studies	AUC (95% CI)	Sensitivity (95% CI)	Specificity (95% CI)	PLR (95% CI)	NLR (95% CI)	DOR (95% CI)
Pleural fluid	12	0.96 (0.94–0.98)	0.94 (0.90–0.96)	0.91 (0.86–0.95)	10.9 (6.4–18.6)	0.07 (0.04–0.12)	157 (57–430)
Blood	4	0.94 (0.92–0.96)	0.92 (0.86–0.95)	0.88 (0.77–0.94)	7.8 (3.7–16.3)	0.10 (0.06–0.16)	81 (27–241)

NT-proBNP: N-terminal pro-brain natriuretic peptide, AUC: area under the receiver operating characteristic curve, 95%CI: 95% confidence interval, PLR: positive likelihood ratio, NLR: negative likelihood ratio, DOR: diagnostic odds ratio

**Fig 3:**
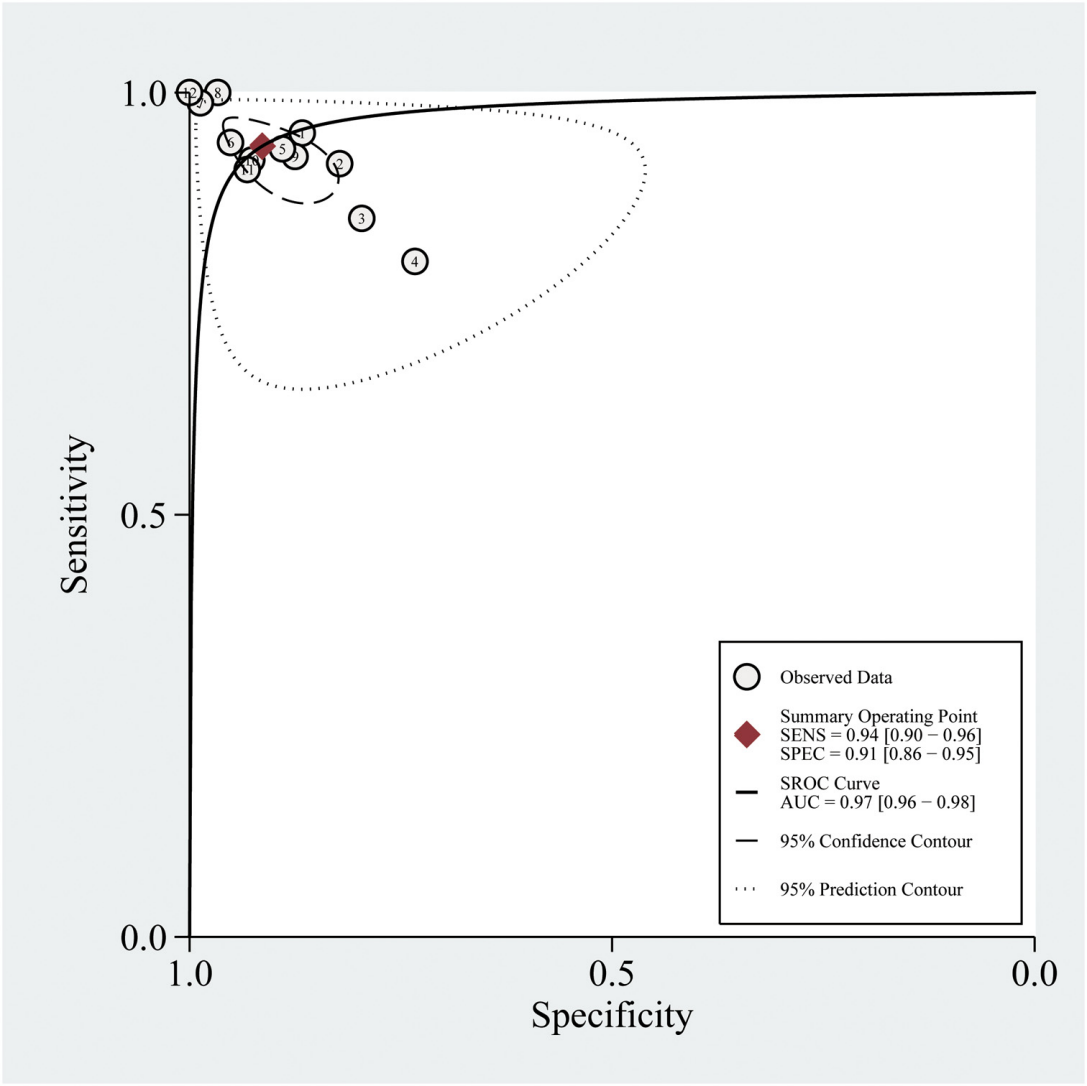
Summary receiver operating characteristic curves for overall diagnostic accuracy of pleural fluid N-terminal pro-brain natriuretic peptide. Each study in the meta-analysis is represented by a solid circle.

The summary ROC (sROC) curve of PF NT-proBNP is shown in Fig 3. The AUC was 0.96 (95% CI: 0.94–0.98). The AUC of the sROC curve for blood NT-proBNP was 0.94 (95% CI: 0.92–0.96).

The pretest probability, which was defined in the present study as the overall prevalence of HF among patients with PF, was 0.36 for PF NT-proBNP and 0.31 for blood NT-proBNP. The post-test probabilities of negative and positive PF NT-proBNP were 0.04 and 0.86, respectively. We also investigated the post-test probability of blood NT-proBNP. The post-test probabilities of negative and positive PF NT-proBNP were 0.04 and 0.78, respectively.

### Publication bias

**Fig 4:**
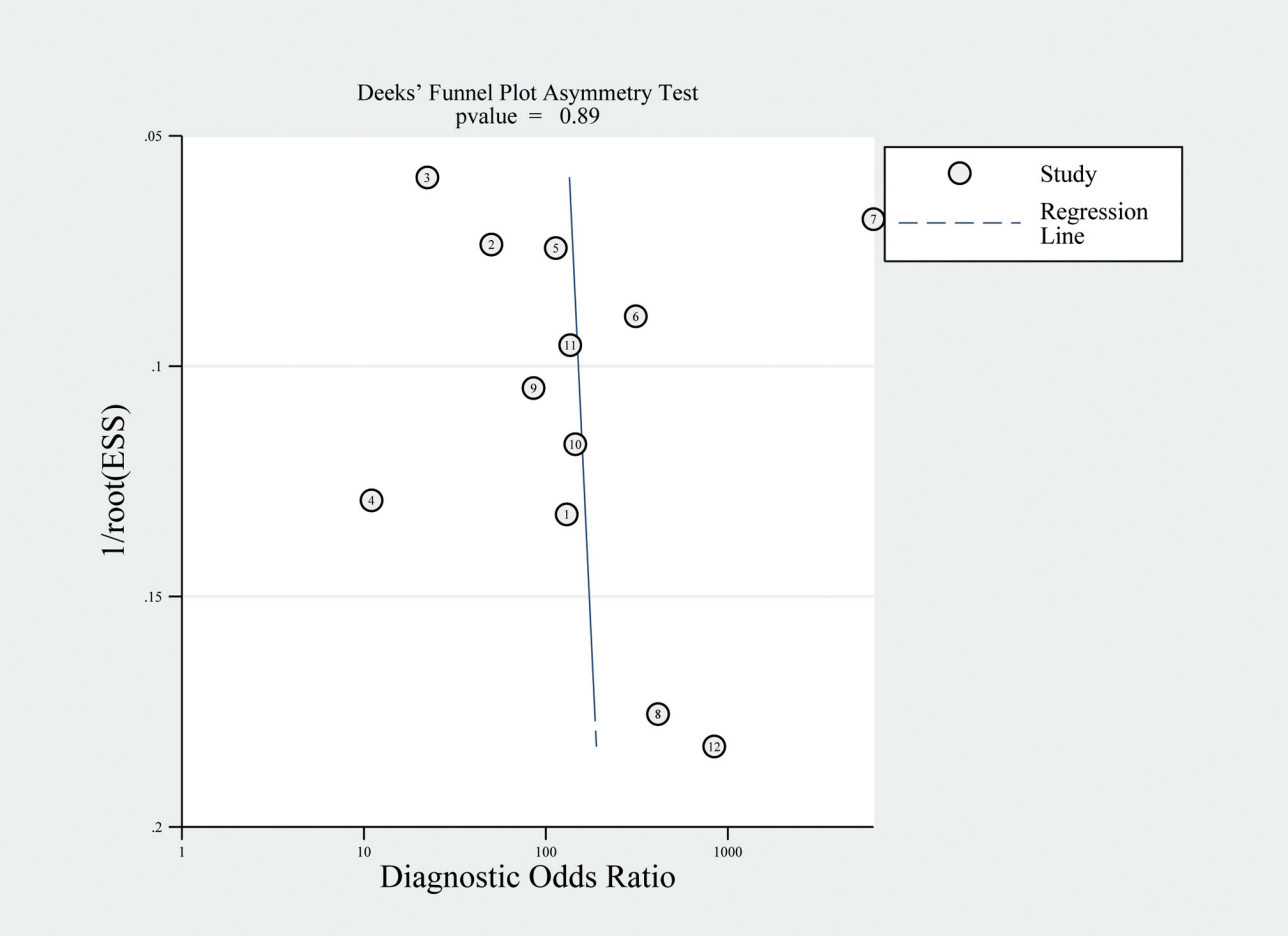
Funnel plot for the assessment of publication bias.

Fig 4 shows a funnel plot for the studies included in the present meta-analysis. Obvious asymmetry was observed. The results of Deeks’ test showed that there was no significant publication bias (P = 0.894) among the 12 studies investigating the diagnostic accuracy of PF NT-proBNP for HF. Publication bias was not assessed for studies that investigated the diagnostic accuracy of blood BNP and NT-proBNP or PF BNP and MR-proANP due to the small number of included studies.

## Discussion

The present systematic review and meta-analysis investigated the diagnostic accuracy of natriuretic peptides for HF in patients with PF. The major findings of the present work are as follows. 1) Both blood and PF NT-proBNP have an extremely high diagnostic value for HF in patients with pleural effusion. 2) PF NT-proBNP has a comparable and overlapping diagnostic value with that of blood NT-proBNP. 3) PF MR-proANP, PF BNP, and blood BNP are effective diagnostic tools for HF, though more studies are needed to rigorously assess their diagnostic accuracy.

Compared with previous meta-analyses on this topic [14, 15, 39], three strengths of the present work should be pointed out. First, because recently published works were included in this systematic review and meta-analysis, the sample size of the present study (*n* = 1654) is larger than that of previous studies (*n* = 907 or 1120), making the results more reliable. Second, in addition to a review of the diagnostic accuracy of PF NT-proBNP, the diagnostic accuracy of PF BNP and MR-proANP and the blood NT-proBNP and BNP were analyzed. Third, in contrast to the traditional sROC approach, which uses the diagnostic odds ratio as the main outcome measure and ignores the tradeoff between sensitivity and specificity, this study utilized a bivariate model, which uses a sensitivity and specificity pair as the starting point of the analysis, and thus may be more reliable for estimating the diagnostic accuracy of the index test in meta-analyses [20]. Additionally, because bivariate models use a random-effects approach for both specificity and sensitivity, any heterogeneity beyond chance could be regarded as a result of clinical and methodological differences among studies. Moreover, the results indicate that, although marked heterogeneity was present across the studies, this heterogeneity was completely attributed to the threshold effect. This indicates that the studies included in the present work were homogeneous. Overall, the results of this meta-analysis are reliable.

In the present meta-analysis, the diagnostic sensitivity and specificity of PF NT-proBNP for diagnosis of HF diagnosis were 0.94 and 0.91, respectively; i.e., 94% of the patients with HF had increased PF NT-proBNP levels and 91% of the patients without HF had decreased PF NT-proBNP levels. Because sensitivity and specificity are often difficult to interpret in clinical practice, positive and negative likelihood ratios (PLR and NLR, respectively) are frequently used because they more directly reflect the clinical utility of a given index test for a target disease. PLR of > 10 or NLR of < 0.10 indicates that the index test is sufficient to verify or exclude the target disease. In the present meta-analysis, the PLR and NLR for PF NT-proBNP were 10.09 and 0.07, respectively, indicating that PF NT-proBNP is sufficient to verify or rule out HF among patients with pleural effusion. The clinical interpretation of blood NT-proBNP is similar to that of PF NT-proBNP. Notably, the PLR and NLR of blood NT-proBNP were 7.8 and 0.10, respectively, indicating that a negative blood NT-proBNP is sufficient to rule out HF while a positive blood NT-proBNP is insufficient to confirm HF.

Because both sensitivity and specificity are affected by the threshold, they might not provide a global view of the diagnostic accuracy of an index test. In contrast, the AUC of the sROC curve is a global index with which to evaluate the diagnostic accuracy of an index test. In the present study, the AUCs of the sROC curves for PF and blood NT-proBNP were 0.96 and 0.94, respectively, indicating that both PF and blood NT-proBNP have high diagnostic accuracy for HF.

Because there are no reliable statistical methods to compare the AUCs of sROCs, it remains unknown whether the AUC for PF NT-proBNP is significantly higher than that of blood NT-proBNP, which would indicate significantly higher overall diagnostic accuracy. Further studies with individual patient data are needed to address this issue. In addition, a high correlation between PF and blood NT-proBNP was reported by three of the eligible studies [27, 30, 35], suggesting that the diagnostic value of blood and PF NT-proBNP for HF may overlap to a large extent. Therefore, it seems that the diagnostic value of PF NT-proBNP for HF diagnosis cannot be improved by blood NT-proBNP, and vice versa. Given that a diagnostic thoracentesis is needed to acquire PF specimens for NT-proBNP determination, blood NT-proBNP remains a preferred choice for diagnosing pleural effusion due to HF. Its high diagnostic accuracy for HF among patients with pleural effusion may therefore eliminate the need for diagnostic thoracentesis [25]. On the other hand, for patients who have undergone diagnostic thoracentesis, PF NT-proBNP determination may help the clinicians to confirm the diagnosis of HF-associated pleural fluids [25].

Some methodological design- and reporting-related weaknesses of the studies in this meta-analysis should be noted to facilitate more optimally designed future studies on this topic. Some of the studies did not enroll patients consecutively [26, 29, 38], leading to potential overestimation or underestimation of the diagnostic accuracy of the index test for the target disease. Ideally, patients should be consecutively enrolled to ensure that the prevalence of the target disease in the patients is as close to that in the real world as possible. Establishment of a prespecified threshold is also important to minimize bias of diagnostic accuracy tests. Data-driven selection of the optimal threshold may introduce bias, especially in studies involving small samples [18]. However, nine studies did not prespecify the threshold [25, 29, 31, 33, 35–38]. Finally, most of the studies in this meta-analysis did not report whether all enrolled patients received the same reference standard. Therefore, neither partial verification bias nor differential verification bias can be completely ruled out. This design weakness may also introduce another type of bias. For example, in one patient with PF, cytologic examination demonstrated malignant pulmonary disease. Therefore, this patient was assigned to the control group. However, because of the lack of a reference standard for this patient, HF could not be completely ruled out, and it may be not reasonable to classify this patient as a control. These design- and reporting-related weaknesses should be avoided in future studies.

The major limitation of the present work is the small number of studies included, particularly the number of studies that investigated the diagnostic accuracy of blood BNP (*n* = 2), PF BNP (*n* = 3), and PF MR-proANP (*n* = 1). Therefore, the diagnostic accuracy of these HF indicators was not analyzed in the meta-analysis. Further studies should be performed to explore this interesting topic.

In conclusion, the present systematic review and meta-analysis found that both blood and PF NT-proBNP are powerful diagnostic indicators of HF in patients with PF. Additional well-designed studies are needed to fully evaluate the diagnostic accuracy of PF and blood BNP and MR-proANP for HF.

## Supporting Information

S1 Checklist PRISMA checklist. (DOC)
